# Drug delivery strategies to cross the blood-brain barrier in Alzheimer’s disease: a comprehensive review on three promising strategies

**DOI:** 10.1016/j.tjpad.2025.100204

**Published:** 2025-05-19

**Authors:** Lotte A. de Koning, Daniel A. Vazquez-Matias, Wissam Beaino, Daniëlle J. Vugts, Guus A.M.S. van Dongen, Wiesje M. van der Flier, Mario Ries, Dannis G. van Vuurden, Everard G.B. Vijverberg, Elsmarieke van de Giessen

**Affiliations:** a Department of Radiology & Nuclear Medicine, Vrije Universiteit Amsterdam, Amsterdam UMC Location VUmc, Amsterdam, the Netherlands; b Cancer Center Amsterdam (CCA), Amsterdam, the Netherlands; c Alzheimer Center Amsterdam, Department of Neurology, Amsterdam Neuroscience, Vrije Universiteit Amsterdam, Amsterdam UMC, Amsterdam, the Netherlands; d Department of Epidemiology & Data Science, Vrije Universiteit Amsterdam, Amsterdam UMC, Amsterdam, the Netherlands; e Center for Imaging Sciences, University Medical Center Utrecht, Utrecht, the Netherlands; f Princess Maxima Center for Pediatric Oncology, Utrecht, the Netherlands; g Amsterdam Neuroscience, Brain Imaging, Amsterdam, the Netherlands

**Keywords:** Alzheimer’s disease dementia, Blood-brain barrier, Brain drug delivery, Focused ultrasound, Receptor-mediated transcytosis, Nanoparticles

## Abstract

The field of Alzheimer’s disease (AD) drug development is rapidly changing, with two anti-amyloid monoclonal antibodies (mAbs) having received Food and Drug Administration (FDA) approval, additionally many compounds are in the pipeline. A major obstacle for novel AD therapeutics is the blood-brain barrier (BBB), which restricts passage of particles larger than 400–500 Da. It is estimated that only ∼0.1 % of mAbs, being ∼150 kDa, passes the BBB, which greatly hampers the efficacy of treatment. To enhance treatment efficacy and to lower the drug dose needed, mechanisms that effectively increase drug delivery across the BBB are urgently sought for. This narrative review describes three promising strategies to enhance drug delivery across the BBB in AD: focused ultrasound (FUS) with microbubbles, receptor-mediated transcytosis (RMT) and delivery using nanoparticle carrier systems. FUS and RMT have shown promising preclinical results and are now being tested in humans whereas nanoparticle carrier systems still need further preclinical validation before clinical application in humans. ^89^Zr-Immuno-PET provides a unique opportunity to noninvasively monitor and quantitatively assess novel brain delivery methods.

## 1 Introduction

### 1.1 Novel therapeutics for Alzheimer’s disease

Alzheimer’s disease (AD) is a neurodegenerative disease characterized by progressive cognitive decline. AD is the most common cause of dementia, followed by vascular dementia, dementia with Lewy bodies, and frontotemporal dementia [1]. Dementia has a profound impact on affected individuals and their caretakers, and on healthcare systems and society as a whole. Due to the aging population, the prevalence of dementia is expected to triple by 2050 [2]. AD drug development has been slow, but in recent years, the field has witnessed a transformative shift with the Food and Drug Administration (FDA) approval of two anti-amyloid monoclonal antibodies (mAbs), Lecanemab and Donanemab, [3,4] and the advent of new types of medication such as anti-sense oligonucleotides (ASOs) and small molecule drugs [5,6]. While mAbs such as Lecanemab and Donanemab represent a promising therapeutic advance by enabling the microglial-mediated phagocytosis of protofibrils and plaques respectively, they are also associated with significant side effects, most notable Amyloid-Related Imaging Abnormalities (ARIA). These side effects have higher incidence in homozygote apolipoprotein (ApoE) ε4 carriers (about 15 % of the AD population), seem to be partly (for ARIA-Edema) dose-dependent and require careful monitoring in clinical use.

A major obstacle for drug development is the blood-brain barrier (BBB), which restricts the passage of molecules larger than 400–500 Da. This is problematic for upcoming disease-modifying therapies (DMTs) such as mAbs and ASOs, which are around 150 kDA and 7 kDA, respectively, and often do not readily cross the BBB. To illustrate, about 0.1 % of mAbs administered dose is estimated to pass the BBB (values reported ranging from 0.01–1 % depending on the measurement approach) [7,8]. Potential mAb candidates have failed, possibly due to crossing the BBB in insufficient quantities to exert a therapeutic effect [9,10]. Mechanisms that safely transport therapeutics across the BBB into the brain can improve effective drug development. Several mechanisms for brain drug delivery have been explored encompassing both invasive (e.g. intrathecal, intracerebral, intraarterial or intraventricular delivery) and non-invasive (e.g. focused ultrasound (FUS), intranasal delivery or delivery using drug-vehicle systems) techniques. Invasive techniques include the risk of adverse side-effects or they show limited spread to distant brain structures. Exploring non-invasive techniques for brain drug delivery therefore warrants further investigation.

**Fig. 1:**
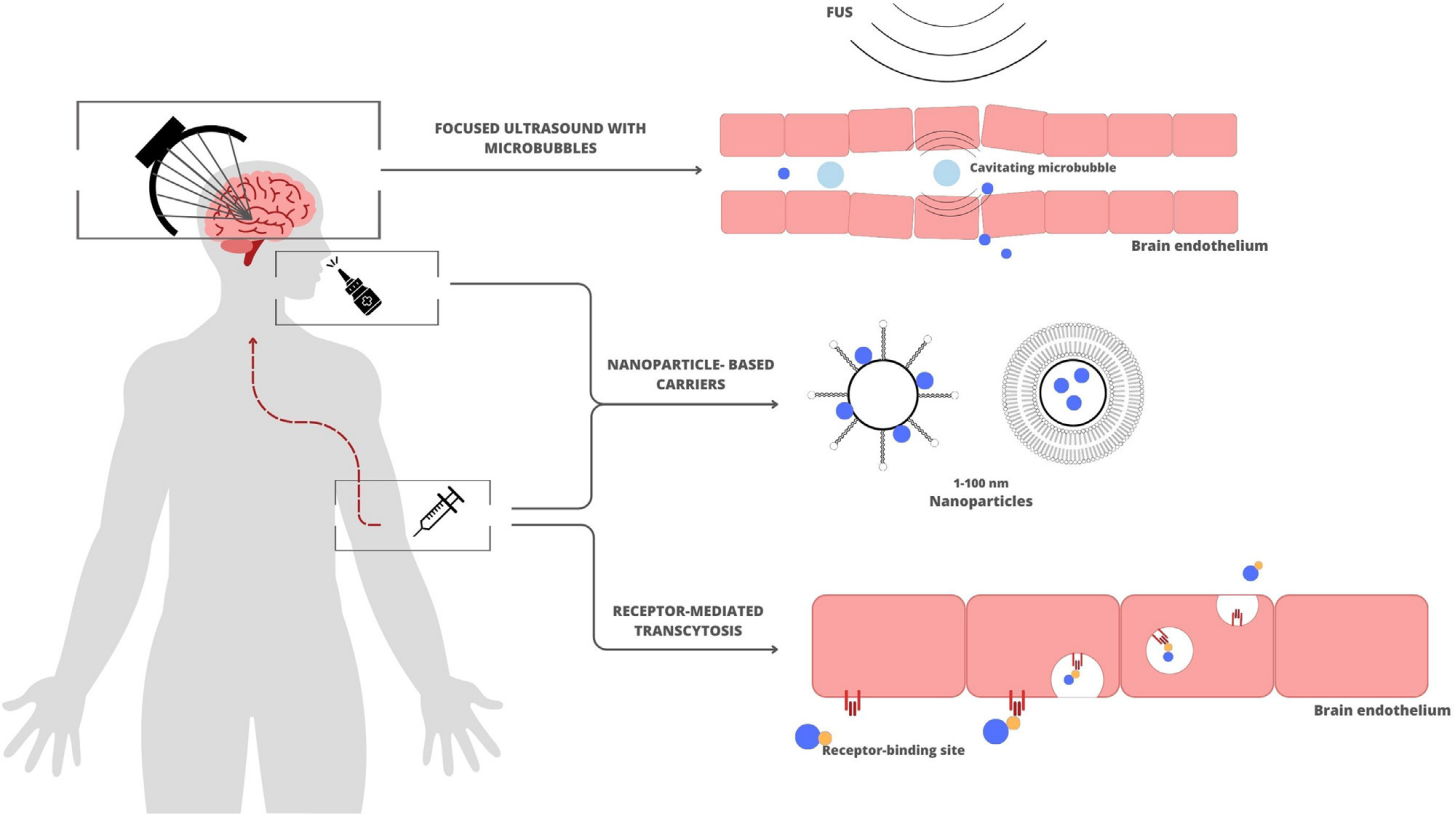
Three approaches to enhance drug delivery in AD dementia. The blue dot indicates AD drug. Nanoparticle illustration © [‘Sara Kerr’] via Canva.com. Other illustrations are found/generated on Canva.com under free content license.

This narrative review provides a comprehensive overview of three current strategies for brain drug delivery in AD dementia (Fig. 1). With recent advances in the field of AD treatment in mind, we aim to highlight two promising strategies (i.e. FUS with microbubbles and receptor-mediated transcytosis (RMT)) for transporting large molecules into the brain. In addition, we explore an upcoming promising strategy utilizing nanoparticle carrier systems for drug transport across the BBB.

### 1.2 BBB physiology, challenges, and possibilities

Structurally, the BBB consists of a tightly interconnected network of endothelial cells lining the capillaries within the brain’s microvasculature. Adjacent capillary and endothelial cells form tight junctions that restrict the passage of most molecules and ions from the bloodstream into the brain tissue. The endothelial cell layer is encompassed by a basal lamina with embedded pericytes. On top, this basal membrane is embraced by astrocyte foot processes. Functionally, the BBB plays a pivotal role in maintaining the brain’s homeostasis by regulating the selective transport of essential nutrients, oxygen, and hormones while preventing the entry of potentially harmful molecules. The BBB is selectively permeable, only allowing molecules with specific properties to pass through. Some small lipophilic molecules can passively enter the brain through paracellular or transcellular diffusion while other molecules pass through by carrier-, receptor-, or adsorptive- mediated transport. This poses a challenge for drug development, as most upcoming DMTs are larger molecules that cannot readily cross the BBB. The exact mechanism by which small amounts of DMTs cross the BBB is not fully understood. Some studies have proposed non-specific endocytosis [11,12] or translocation across the blood-cerebrospinal fluid barrier [13] as potential mechanisms by which for example mAbs may enter the brain. Even if a drug succeeds in crossing the endothelial cell membrane, it can encounter ATP-binding cassette (ABC) transporters on the luminal and abluminal side of the brain endothelium that actively pump compounds back into the blood stream [14]. In addition, besides other factors like aging that influence BBB permeability [15], AD heterogeneity can also contribute to varying BBB permeability, with certain AD subtypes exhibiting a dysfunctional BBB [16,17]. The underlying pathophysiological process behind BBB dysfunction in AD remains poorly understood. Several factors like amyloid pathology, vascular damage, neuroinflammation and ApoE genotype may influence BBB integrity but it remains unclear whether alterations in BBB integrity facilitate or impede the penetration of therapeutic molecules into the brain [17,18]. It is possible that loss of BBB integrity causes leakiness facilitating the entry of antibodies, but it is also possible that blood-derived debris and cells accumulate in enlarged perivascular spaces, ultimately limiting large therapeutic molecules from entering the brain [18].

There are several methods to increase drug delivery into the brain. First, several ways can be exploited to open up the BBB tight-junctions. Examples of this are FUS with microbubbles and intra-arterial injection of mannitol. Intra-arterial mannitol injection has been widely used in cancer research and facilitates osmotic opening of the entire BBB, in contrast with FUS, which facilitates local targeted BBB opening. In addition, the biochemical properties of a drug can be altered in such a way that they can readily cross the BBB. A technique for this could be by attaching a transporter protein to the drug that facilitates transport across the BBB through RMT. Third, therapeutics can be loaded into (or attached to) nanoparticle-based carriers (e.g. extracellular vesicles, liposomes, polymeric nanoparticles), which improve BBB permeability. Finally, drugs can physically bypass the BBB by adjusting the routes of administration (i.e. intraventricular, intrathecal, and intranasal administration). Intrathecal administration is used for delivery of drugs through the spinal cord into the cerebrospinal fluid (CSF), but it is invasive and does not always result in uniform drug distribution [19]. It is thought that intranasal administration allows drugs to bypass the BBB as they pass through the olfactory pathway. However, drugs have to pass through the nasal canal, which restricts dosing capacity. Intranasal delivery of small DMTs, such as insulin, has been investigated, but intranasal delivery of large DMTs is still poorly understood.

## 2 Current strategies for brain drug delivery in AD

### 2.1 Focused ultrasound (FUS) with microbubbles

FUS was first reported in 1942 [20] and has a wide range of applications, including the treatment of essential tremor in Parkinson’s disease [21,22]. Low-intensity FUS, when combined with microbubbles, can induce temporary and reversible disruptions in the BBB by opening tight junctions in a targeted area, allowing for the passage of large therapeutic molecules. In addition, FUS BBB opening is thought to enhance transcellular drug transport and influence ABC transporter expression and functionality, although the extent of reduced functionality remains to be seen [23]. Recent studies furthermore evidenced the generation of pores and tunnels in endothelial cells providing gateways into the parenchyma [24]. The advantages of FUS BBB opening are its spatial (mm accuracy) and temporal accuracy. Given its ability to reversibly open the BBB, FUS presents a promising perspective for brain drug delivery. A schematic overview of the reviewed preclinical and clinical publications regarding FUS can be found in Supplementary Table 1.

**Preclinical research**

FUS has been safely applied to preclinical AD models, including rodents [[25], [26], [27], [28]]. In addition, the safety of neuronavigation-guided FUS has also been tested in non-human primates [29]. Even in the absence of therapeutic intervention, FUS BBB opening has shown promising results for AD. Several preclinical studies investigating the effects of FUS BBB opening have shown that FUS alone can reduce amyloid pathology in AD mouse models[25,28,[30], [31], [32]]. The mechanisms behind FUS-induced amyloid reduction in mice are not yet fully understood, but it is thought that FUS BBB opening promotes microglial cell activation and subsequent internalization of amyloid plaques [25,30]. It is also possible that FUS causes extravasation of plasma proteins (e.g. albumin), and thereby activates astrocytes and microglia [33]. In addition, increased levels of endogenous immunoglobulins are thought to enter the brain and co-localize near amyloid plaques thereby suggesting a possible relationship with FUS-mediated removal of amyloid plaques [25]. Similarly, transgenic AD tau mouse models show bilateral reductions in phosphorylated tau in response to unilateral FUS BBB opening. Interestingly, these reductions are accompanied by increased immune response in both the FUS-targeted as well as the contralateral control hemisphere compared to control brains [34]. Although the immediate effect of FUS-mediated amyloid removal seems beneficial, repeated activation of microglia can lead to sustained neuroinflammation and subsequent disease deterioration. More research is therefore urgently needed to fully understand the mechanisms behind FUS-induced amyloid reduction and to investigate the clinical effect of repeated treatments.

To enhance drug delivery across the brain and improve treatment efficacy, preclinical studies have applied FUS BBB opening in conjunction with DMTs such as anti-amyloid [26,[35], [36], [37], [38]] and anti-tau [[39], [40], [41]] mAbs, glycogen synthase kinase-inhibitor (GSK) [42], intravenous immunoglobulin (IVIg) [43], and tropomyosin receptor kinase A (TrkA) agonist [44] delivery. Microbubble-mediated scanning ultrasound (SUS), ultrasound that does not target a specific area but is rather moved across the skull, increases levels of a full sized or single chain tau antibody (by 19- (measured one hour after one treatment) or 11-fold (measured 30 min after four treatments)) [39,41] and an anti-amyloid antibody (by 5-fold, three days after nine SUS treatments) in the brain [36]. However, it is still largely unknown whether increased antibody brain penetrance also leads to enhanced tau and amyloid clearance and, consequently, a therapeutic benefit in humans. In a preclinical AD mouse model, microbubble-mediated MRI-guided FUS (MRgFUS) BBB opening in combination with the administration of anti-amyloid mAb leads to a 23 % reduction in overall amyloid plaque surface area [26], whereas FUS BBB opening by MRgFUS alone (i.e. without intravenously administered anti-amyloid mAbs) leads to a 13 % reduction after four days of treatment [25]. Similarly, FUS BBB opening by microbubble-mediated SUS alone has been shown to be sufficient in removing amyloid plaques and restoring cognitive function [30]. Thus, the question remains what percentage of amyloid plaque removal can be attributed to FUS mediated factors (such as the influx of immunoglobulins) and what is removed due to the therapeutic effect of anti-amyloid mAbs. Additional preclinical evidence suggests that, increased anti-amyloid antibody brain penetrance in mice results in increased amyloid clearance in the cortex and improved cognitive outcomes [36]. Recent results in APP23 mice suggest that cognitive improvement can occur following high frequency (1 MHz) SUS treatment, in absence of microbubble-induced BBB opening and subsequent amyloid removal [45].

**Clinical research**

In clinical studies, current FUS devices for BBB opening can be subdivided into three main types: implanted ultrasound devices, neuronavigation-guided devices, and MRgFUS, of which MRgFUS has been used most in the context of AD. MRgFUS combines FUS with real-time magnetic resonance imaging guidance. The first human clinical trial incorporating microbubble-mediated MRgFUS in five AD dementia patients showed the technology to safely and efficiently open the BBB in the right frontal lobe [46]. In this study, BBB opening and closing is determined using gadolinium-contrast enhanced T1 MRI, and safety is shown by the absence of clinical or radiographic adverse events [46]. The safety of MRgFUS targeting other brain regions has since then been replicated in other studies in both AD [47,48] and Parkinson’s disease dementia [49,50]. Park and colleagues were the first to safely incorporate MRgFUS-BBB opening in more extensive brain regions (i.e. two FUS sessions targeting bilateral frontal lobes) [47], covering a mean volume of about 21 cm^3^. FUS-targeted brain regions have since then been extended to frontal, parietal and medial temporal lobes (three FUS sessions) [51], covering about 30 cm^3^. Gadolinium enhanced MRI sequences indicate that the BBB opening is reversed 24–48 h after each FUS treatment [46,48,51]. Secondary clinical outcomes (i.e. changes in amyloid pathological load and cognition) of MRgFUS mediated BBB opening have shown mixed results. Some clinical trials (five to six participants in each trial) show that MRgFUS by itself can induce relative amyloid plaque reductions following two [47] and three FUS sessions [52], while other studies see no changes on amyloid-PET five weeks after the first FUS treatment [46]. In addition, although preclinical studies on transgenic mice indicate cognitive improvements following FUS BBB opening [28,31], clinical studies indicate no change in the rate of cognitive decline [47,51,53]. One clinical trial indicates mild cognitive improvements are seen shortly after sonication in the right hippocampus, even though BBB opening was not achieved [54]. BBB opening using other FUS devices (i.e. implantable or neuronavigation-guided FUS) has been less extensively studied, but an implantable ultrasound device (SonoCloud-1, Carthera, Paris, France) has recently been tested for safety in humans and did not show any significant changes in amyloid- or FDG-PET post-treatment [55].

An ongoing clinical trial is investigating the utility of monthly MRgFUS BBB opening in conjunction with Aduhelm® (Aducanumab) infusion therapy (up to six sessions with Aduhelm® doses from one to six mg/kg) in patients with mild cognitive impairment or mild dementia due to AD [56]. The first three patients have undergone six cycles of monthly Aduhelm® infusion with FUS BBB opening, and have shown significant amyloid reductions in the FUS-treated regions compared to the contralateral brain regions [57].

### 2.2 Receptor-mediated transcytosis (RMT)

A second strategy for brain drug delivery is re-engineering therapeutic antibodies into bispecific antibodies by fusing them with transporter moieties that binds to a receptor expressed on the BBB endothelium and shuttles the therapeutic antibody across the BBB. Binding of the transporter antibody to the membrane-bound receptor of interest enables a process called receptor-mediated transcytosis (RMT) to transport the bispecific antibody across the BBB [58]. Upon binding, the antibody-receptor complex is internalized via endocytosis, forming a vesicle that traverses the endothelial cell. The antibody is then released into the brain parenchyma through exocytosis on the abluminal side. This process is influenced by several factors such as binding affinity of the bispecific antibody, with high affinity potentially hindering antibody release into the brain, and intracellular pH, which regulates lysosomal trafficking and promotes mAb degradation under acidic conditions [59]. In addition, RMT with transporter ligands can be exploited to transport other therapeutic molecules, such as oligonucleotides [60,61] and enzymes [[62], [63], [64]] across the BBB in AD dementia and other neurological disorders. A schematic overview of the reviewed preclinical and clinical sources regarding RMT can be found in Supplementary Table 2.

**Preclinical research**

In AD, a range of therapeutic antibodies has been engineered into bispecific antibodies by conjugating them with transporter moieties targeting one of two main receptors: transferrin receptor 1 (TfR1), which in humans normally binds iron-carrying transferrin particles and is highly expressed on brain endothelial cells [65], and the insulin receptor (IR) [66]. Preclinical research has shown that bispecific antibodies that bind both (murine/human) amyloid and murine TfR increase antibody brain uptake in AD transgenic mice by 7–50 fold [[67], [68], [69], [70], [71]]. Secondary outcomes on subsequent amyloid reduction are inconclusive; some studies show decreases in the total number of plaques [70] and in soluble amyloid protofibrils following administration of a bispecific antibody [72], whereas another study implies no effects on soluble amyloid protofibrils upon increased mAb brain exposure [73]. The absence of a reduction in soluble amyloid protofibrils could be attributed to low baseline levels of soluble amyloid protofibril in the App*^NL−G−F^* mouse model [73]. Preclinical research investigating BBB shuttles has also extended to non-human primates. They show increased central nervous system (CNS) uptake following peripheral administration of IR-, and TfR-targeted BACE1 [74,75] by 30-fold [74] and amyloid bispecific antibodies [76,77] by 4–18 fold [76]. Similar brain shuttle mechanisms exploiting TfR-mediated transcytosis have been utilized in other preclinical studies for delivery of the amyloid-degrading enzyme Neprilysin [62,63] and a TREM2 activating antibody [78], where they show that (murine [62] and human [78]) TfR-functionalized brain shuttles show increased brain concentration by about 2- to 6-fold for a TREM2 antibody and 20-fold and Neprilysin. Another potential BBB target for AD drug delivery to the brain is CD98hc (the heavy chain of amino acid transporter LAT1). Preclinical studies using BACE1/CD98hc and TrkB/CD98hc bispecific antibodies show greater increases in brain concentration using CD98hc shuttles compared to TfR-shuttles, possibly due to longer antibody brain exposure [79,80].

**Clinical research**

There is currently one ongoing clinical trial investigating the use of TfR1-mediated drug delivery in Alzheimer’s disease (i.e. NCT04639050, Roche). Roche’s Trontinemab (Gantenerumab fused to a monovalent anti-human TfR1-binding Fab fragment) has shown to be safe in healthy individuals and has recently entered Phase II testing in AD patients [81,82]. First results show rapid reductions on amyloid-PET at lower dose levels than with typical anti-amyloid mAbs [82]. In addition, Trontinemab results in 8-fold higher CSF to plasma ratios [83,84] and in far lower ARIA rates compared to classical IgG mAbs. It is thought that the TfR1-mediated drug delivery causes faster widespread delivery to the brain through smaller TfR-coated vessels, bypassing large artery amyloid depositions [85]. This could be a potential reason for the low ARIA rates seen in early results of the phase I/II trial of Trontinemab, which is particularly promising. Another Phase I study (i.e. NCT05450549, Denali) investigating a bispecific TfR1-TREM2 antibody was recently strategically halted by the leading companies after moderate hematological changes were detected at high drug doses suggesting a small therapeutic window [86].

Clinical studies should consider several factors to optimize drug delivery through RMT. For example, important considerations that can affect the effectiveness of antibody’s brain penetrance are the antibody dose [68] and the type of binding between the transporter antibody and the endothelium target receptor. It has been reported that monovalent, medium-affinity binding between the anti-TfR antibody and the TfR results in improved BBB transport [70,87]. Low affinity binding reduces binding to the TfR on the BBB and subsequent brain exposure, whereas high affinity binding between the anti-TfR antibody and the TfR promotes lysosomal degradation. Moreover, the potential of peripheral sink effects should be taken into account. For example, TfR1 is also expressed in the spleen and bone marrow and can therefore cause off-target sequestration [88]. The expression of TfR1 on immature red blood cells called reticulocytes also raises the risk of off-target binding by bispecific antibodies, potentially leading to reticulocyte depletion and subsequent anemia [89]. In contrast with FUS-mediated BBB opening, RMT through bispecific antibodies lacks the capability for local delivery to a selected brain region. Finally, AD dementia is associated with altered BBB function, and it is yet unknown how this influences RMT-exploiting transport mechanisms.

### 2.3 Nanoparticle-based carriers

Nanoparticle-based carriers are 1–200 nm in size and have favorable physicochemical properties for crossing the BBB. They can be loaded with or attached to macro- and small molecular drugs to allow for increased brain drug delivery. The utility of nanoparticle-based carriers for brain drug delivery in AD has been explored in vitro and in preclinical in vivo studies. In human research, nanoparticles have shown promise as carriers for intranasal administration of drugs. It is thought that intranasal administration allows drugs to physically bypass the BBB as they pass through the olfactory pathway. An overview of the reviewed preclinical and clinical sources regarding nanoparticle-based carriers can be found in Supplementary Table 3.

**Preclinical research**

Several nanoparticles have been explored in vitro and in vivo for drug delivery in AD dementia. Examples are liposomes, solid-lipid nanoparticles (SLNPs), polymeric nanoparticles, metal nanoparticles, and extracellular vesicles. The physicochemical properties of these nanoparticles, including their shape, size, and zeta potential (surface charge), must be carefully optimized to enhance their efficacy and minimize cytotoxicity. These parameters also play a crucial role in drug loading capacity, entrapment efficiency, and release profile, all of which are critical for effective brain drug delivery. A high loading capacity enables each nanoparticle to carry a greater amount of drug, which is particularly important for crossing the BBB where only a small fraction of the administered dose typically reaches the CNS. Low drug loading capacity not only raises drug cost, but more importantly results in suboptimal release patterns, ultimately limiting the overall drug release. Moreover, a controlled release profile helps maintain consistent drug levels in the brain over time, enhancing therapeutic efficacy. In addition, nanoparticles are often coated with specific targeting ligands to improve circulation time and enhance transport across the BBB. In vitro evidence shows that penetration across human cerebral microvascular endothelial cells (hCMEC/D3, an often used BBB model) may differ across different types of lipid nanoparticles (SLNPs versus nanostructured lipid carriers, where nanostructured lipid carriers are thought to have improved stability and loading capacity over SLNPs) [90], with higher permeability found across nanostructured lipid carriers. Interestingly, the researchers also show that TfR-functionalization does not significantly increase hCMEC/D3 cell penetration for either type of lipid nanoparticle, possibly due to saturation of TfR-binding sites at the BBB. In addition, no cytotoxicity was seen on hCMEC/D3 cells, however it is important to validate these findings on other cell lines. Other in vitro evidence demonstrates that ApoE-functionalization of SLNPs carrying donepezil enhances permeability across a co-culture BBB model by 3-fold compared to non-functionalized SLNPs [91]. In this study, functionalization using ApoE results in a decreased encapsulation efficiency of donepezil. In line with this, nanoparticle functionalization has shown promising effects in preclinical animal models of AD in which delivery of anti-amyloid heavy chain antibody fragments (VHH) is increased by intravenous injection of glutathione- and PEG-coated (addition of polyethylene glycol to prolong blood circulation time) liposomes compared to a non-liposomal VHH [92]. Another study shows that nanoparticle functionalization with polysorbate 80 (thought to induce RMT through low-density lipoprotein receptor-related protein binding) is necessary to increase brain transport of rivastigmine-loaded nanoparticles [93]. It shows that only polymeric nanoparticles coated with polysorbate 80 increase brain concentrations of rivastigmine by 3.8-fold, whereas non-functionalized nanoparticles do not increase brain concentrations of rivastigmine in wild-type rats [93]. It also shows that polysorbate 80 coating slightly reduces the drug loading of nanoparticles, and subtly changes the zeta potential. Preclinical studies also report improvement of cognition following polymeric nanoparticles functionalized with an amyloid antibody [94] and reduction of neuroinflammation following lipid nanoparticles carrying anti-17 (inhibiting miR-17) and functionalized with mannose (to target delivery to microglia) [95]. Overall, preclinical animal models support the use of nanoparticle drug delivery for Alzheimer’s disease [96]. Preclinical evidence suggests liposomes, polymeric nanoparticles and SLNPs can be suitable candidates for drug delivery in AD, and their effects can be enhanced by nanoparticle functionalization. However, toxicity can be a concern with nanoparticle delivery systems and more preclinical research is needed before they can make it into a clinical setting. Factors such as size, generally thought to be inversely related to cytotoxicity, zeta potential, and nanoparticle coating are thought to influence cytotoxicity and should therefore be carefully investigated.

Nanoparticles have also been investigated for the delivery of drugs through the intranasal route. With intranasal administration, drugs typically have to pass the mucus layer and cross the nasal epithelium, after which they travel via the olfactory and trigeminal nerves to the olfactory bulb, brainstem and the rest of the CNS. Two studies using wild-type rats and rabbits demonstrated that intranasal administration of donepezil using liposomes increased donepezil brain delivery more than 2-fold compared to oral administration [97,98]. Given the local delivery through the nasal canal, it is still a question to what extent drugs can penetrate more distant brain regions after intranasal administration. Moreover, translation from preclinical to clinical setting is challenged by differences in the anatomy of nasal mucosa between species with large differences in olfactory epithelium and mucociliary clearance rates [99].

**Clinical research**

The clinical application of nanoparticles to enhance AD drug delivery is limited. Nanoparticles play a large role in vaccinology for other diseases [100], but until now, AD research has mainly focused on utilizing nanoparticles as vehicles for intranasal drug administration. An ongoing phase-II clinical trial in AD patients utilizes nanoparticles for intranasal delivery of an alpha-secretase modulator [101]. Limiting factors for clinical application of intranasal delivery are the volume and bioavailability of drugs, with hydrophilic drugs showing poor absorption. Other nanoparticle applications are still in preclinical stages and need further validation before clinical validation can take place.

## 3 Discussion and future directions

The AD drug development pipeline faces numerous challenges, and one of the primary obstacles is the ability of the drug to reach the brain. As the first generation of DMTs shows clinical promise, it becomes crucial to explore mechanisms that can enhance drug efficacy. Increasing brain penetration emerges as a critical factor in this pursuit. Strategies to enhance drug delivery such as FUS with microbubbles and RMT provide promising methods to increase brain drug delivery, while other methods such as nanoparticle-based carriers need more widespread preclinical validation before clinical application can take place.

FUS BBB opening has high spatial precision and can therefore be used to target distinct brain regions. This is relevant in the early disease stage when limited brain regions are affected and for the variability in the spatial distribution of brain pathology among AD patients. However, given the diffuse nature of amyloid and tau pathology in later stages of the disease, a strategy that ensures both spatial precision and broad coverage is required. Repeated and extensive FUS treatments across multiple brain regions may be required, which can increase procedure time, patient burden, and logistical complexity. This raises concerns regarding the feasibility, cost-effectiveness and safety of this technique. Although FUS-mediated BBB opening allows passage of not only administered drugs but also of other circulating endogenous compounds, the first clinical trials support the safety of this technique. To further demonstrate clinical safety and feasibility, the optimal FUS parameters (e.g. optimal number and volume of FUS sessions and microbubble properties) must be determined. Moreover, the first clinical results suggest that combined therapy of antibody delivery and FUS BBB opening results in a larger reduction in amyloid-PET burden, though the extent of increased antibody delivery to the brain remains unclear. BBB dysfunction found in a subset of AD patients can affect therapeutic brain penetrance, and it is therefore important to understand what percentage of amyloid reduction can be attributed to increased drug delivery caused by FUS. Gadolinium contrast application can indicate BBB opening, but direct quantitative measures of mAb delivery to the brain, using for example PET-imaging, are limited until now. In a recent oncology study, researchers utilize ^111^In-radiolabed trastuzumab to evaluate and quantitate the brain delivery of mAb trastuzumab following FUS BBB opening by SPECT-imaging. Their findings reveal a 2-fold increase in the accumulation of radiolabeled trastuzumab within MRgFUS-treated lesions [102], highlighting the promise of FUS BBB opening for mAb delivery to the brain. Caution is warranted in interpreting these quantitative findings as brain metastases are characterized by fenestrated endothelium and may therefore not be representative of other neurological disorders.

RMT facilitates more widespread drug delivery to the brain and the first clinical results using RMT to enhance drug brain penetrance in AD show that it can increase CSF to plasma ratios by 8-fold compared to typical mAbs. RMT may lead to greater increases in drug delivery to the brain, however, in order to facilitate direct comparisons of results, direct measures of drug delivery to the brain in neurodegenerative disorders such as AD are necessary. The impact of AD heterogeneity on drug delivery via RMT and nanoparticle-based carriers remain poorly understood. Notably, vascular comorbidities commonly observed in AD can lead to brain endothelium degeneration and reduced BBB transporter expression, such as TfR1, thereby compromising BBB integrity and limiting the efficiency of therapeutic delivery. More research is needed to clarify how the pathological heterogeneity of AD, particularly differences in vascular integrity and presence of other copathologies, impacts the safety, efficiency, and targeting of FUS, RMT, and nanoparticle-based carrier delivery systems.

Despite the various strategies developed to enhance drug delivery to the brain, accurately quantifying the amount of drug that reaches the brain tissue remains a significant challenge. Existing techniques, gadolinium-enhanced MRI and CSF to plasma ratios, provide indirect evidence of drug delivery but fall short of offering precise quantification. One promising approach to address this need is the use of ^89^Zr-immuno-PET imaging. By labeling mAbs with ^89^Zr, it becomes possible to non-invasively visualize and measure the amount of drug that penetrates the BBB and accumulates in brain tissue for several days. This technique provides a direct and quantitative assessment of drug delivery, which is crucial for optimizing therapeutic strategies and determining necessary drug dosages for effective treatment. ^89^Zr-immuno-PET has already been applied in numerous oncological studies [103]. Translation and application of ^89^Zr-immuno-PET to neurodegenerative diseases such as AD is vital to better understand the biodistribution and efficacy of emerging mAbs. Its clinical application, however, requires highly sensitive total body PET-CT scanners to minimize radiation burden. In addition, ^89^Zr-immuno-PET can facilitate the evaluation of the efficacy of the novel drug delivery methods like FUS and RMT. Recently, a clinical trial was initiated to evaluate the biodistribution of ^89^Zr-labeled Trontinemab (EudraCT No. 2023–509,900–14–00) [104], highlighting the potential role of ^89^Zr-immuno-PET in guiding and optimizing novel drug delivery methods.

## Funding

This research was funded by a grant from the Hersenstichting (Dutch Brain Foundation) to the Dutch Neuro FUS consortium (DR-2021-00391).

## CRediT authorship contribution statement

**Lotte A. de Koning:** Writing – original draft, Conceptualization. **Daniel A. Vazquez-Matias:** Writing – review & editing. **Wissam Beaino:** Writing – review & editing. **Daniëlle J. Vugts:** Writing – review & editing. **Guus A.M.S. van Dongen:** Writing – review & editing. **Wiesje M. van der Flier:** Writing – review & editing, Conceptualization. **Mario Ries:** Writing – review & editing. **Dannis G. van Vuurden:** Writing – review & editing. **Everard G.B. Vijverberg:** Writing – review & editing, Conceptualization. **Elsmarieke van de Giessen:** Writing – review & editing, Supervision, Conceptualization.

## Appendix Supplementary materials

Image, application 1
