# Supplementary material for: Drug delivery strategies to cross the blood-brain barrier in Alzheimer’s disease: a comprehensive review on three promising strategies
Source: J Prev Alzheimers Dis. 2025 May 19;12(7):100204. doi: 10.1016/j.tjpad.2025.100204 (PMC12321624; doi:10.1016/j.tjpad.2025.100204)
Supplement: Supplementary file 1 [file mmc1.docx]

**Supplementary Table 1**: Summary of the reviewed sources for section 2.1 Focused ultrasound (FUS) with microbubbles

| **Authors** | **Method** | **#Treatments** | **Drug type** | **Population** | **Results** |
| --- | --- | --- | --- | --- | --- |
| Jordão et al (2013) | MRgFUS | One session | / | TgCRND8 mice | Increases endogenous antibodies in FUS-treated regions and glial cell markers and reduces Aβ plaques |
| Jordão et al (2010) | MRgFUS | One session | Anti-Aβ antibody | TgCRND8 mice | Reduces Aβ plaques |
| Choi et al (2008) | FUS | Twice 30s sonication | / | APP/PS1 mice | BBB opening and closing differs per brain region targeted |
| Burgess et al (2014) | MRgFUS | Four weekly sessions | / | TgCRND8 mice | Improves cognition and reduces Aβ plaque load following bilateral FUS treatment |
| Pouliopoulos et al (2021) | Neuronavigation-guided FUS | One session | / | Rhesus macaques | Neuronavigation is safe and triggers a temporary immune response |
| Leinenga et al (2015) | SUS | Five sessions in six weeks | / | APP23 mice | Reduces Aβ plaques and improves memory performance on three tasks |
| Karakatsani et al (2023) | Single-element FUS transducer | Four weekly sessions | / | 3xTg-AD mice | Reduces both tau and Aβ pathology following bilateral FUS; improves cognition; cholesterol metabolism-associated genes are upregulated |
| Poon et al (2018) | MRgFUS | Three-five biweekly sessions | / | TgCRND8 mice | Reduces Aβ plaques following bilateral FUS treatment |
| Karakatsani et al (2019) | Single-element FUS transducer | Four weekly sessions | / | rTg4510 mice | Unilateral treatment reduces bilateral phosphorylated tau |
| Kong et al (2022) | Single-element FUS transducer | Three biweekly sessions | Anti-Aβ antibody (Aducanumab) | 5xFAD mice | Increases antibody delivery into the brain; combined treatment resulted in less cognitive decline and larger Aβ plaques reduction |
| Leinenga et al (2021) | SUS | Four weekly sessions, followed by five sessions in 27 weeks | Anti-Aβ antibody (Aducanumab analog) | APP23 mice | SUS increases antibody delivery; there is an additive effect of combined SUS/antibody treatment on plaque reduction in cortex, but not in hippocampus |
| Antoniou et al (2023) | Single-element FUS transducer | One session | Anti-Aβ antibody | 5xFAD mice | Increases antibody delivery to the brain |
| Bathini et al (2022) | FUS | One session | Anti-Aβ antibody | APP/PS1 mice | Increases antibody brain delivery with activated microglia and causes transient neutrophil infiltration |
| Nisbet et al (2017) | SUS | Four weekly sessions | Anti-tau antibody | pR5 mice | Increases antibody brain uptake and efficacy |
| Bajracharya et al (2022) | SUS | 12 weekly sessions | Anti-tau antibody | K3 mice mice | Increases antibody brain delivery; but no enhanced therapeutic effect |
| Janowicz et al (2019) | SUS | One session | Anti-tau antibody | pR5 mice | Larger, full sized IgG (compared to smaller antibody fragments) results in higher brain uptake |
| Hsu et al (2008) | FUS | Five weekly sessions | GSK-3 inhibitor | APP/PSEN1 mice | Results in increased reduction of GSK-3 activity and plaque reduction |
| Dubey et al (2020) | MRgFUS | Two sessions in eight days | IVIg | TgCRN8 mice | FUS increases brain delivery of IVIg. Combined treatment (as well as FUS and IVIg separate) reduces Aβ. Combined treatment significantly increases hippocampal neurogenesis |
| Xhima et al (2022) | MRgFUS | Three weekly sessions | TrkA agonist | TgCRND8 mice | Combined (with bilateral FUS) treatment improves memory; promotes cholinergic activity; reduces plaques; enhances hippocampal neurogenesis |
| Leinenga et al (2024) | SUS | Eight weekly sessions | / | APP23 mice | (High frequency) SUS (without microbubbles, BBB opening, and amyloid removal) results in functional connectivity changes and improved memory |
| Lipsman et al (2018) | MRgFUS | Two monthly sessions | / | Mild-to-moderate AD | MRgFUS safely and transiently opens the BBB; no changes on Aβ-PET and no worsening of cognitive decline |
| Park et al (2021) | MRgFUS | Two sessions three months apart | / | Moderate-to-severe AD | Small decreases in Aβ-PET; no difference in cognition |
| Rezai et al (2020) | MRgFUS | Three sessions three weeks apart | / | Mild AD | MRgFUS safely and transiently opens the BBB in hippocampus/entorhinal cortex |
| Gasca-Salas et al (2021) | MRgFUS | Two sessions two-three weeks apart | / | PDD | Results in slight cognitive improvements and no changes in Aβ or fluorodeoxyglucose (FDG) PET |
| Pineda-Pardo et al (2022) | MRgFUS | Two sessions two-four weeks apart | / | PDD | MRgFUS safely and transiently opens striatal BBB; no changes in motor score; decreases Aβ-PET but no change FDOPA PET |
| Karakatsani et al (2023) | Neuronavigation-guided FUS | One session | / | Mild AD | N=1 patient shows a non-persistent reduction on Aβ-PET |
| Rezai et al (2023) | MRgFUS | Three sessions, each two weeks apart | / | Mild AD | Decreases in Aβ-PET; no worsening cognitive decline |
| D’Haese et al (2020) | MRgFUS | Three sessions, each two weeks apart | / | Mild AD | Decreases in Aβ-PET in FUS-treated regions; 2/6 patients saw reduction in Aβ on contralateral side |
| Meng et al (2023) | MRgFUS | Three biweekly sessions | / | Mild-to-moderate AD | Decreases in Aβ-PET; no changes in CSF or plasma biomarkers; no worsening of cognitive decline |
| Jeong et al (2021) | Image-guided FUS | One session | / | Moderate to severe AD | No BBB opening; decreases on FDG-PET; subtle cognitive improvement after short follow-up |
| Epelbaum et al (2022) | Implantable FUS | Seven sessions each two weeks apart | / | Mild AD | Implantable FUS is safe; no significant changes in Aβ-PET, glucose metabolism or cognition |
| Rezai et al (2024) | MRgFUS | Six monthly sessions | Aducanumab | MCI due to AD or mild AD | Combined treatment results in increased Aβ removal |
| Meng et al (2021) | MRgFUS | Up to six sessions | Trastuzumab | Patients with Her2-positive breast cancer and brain metastases | Increases in antibody delivery to brain metastasis |

Preclinical/animal research and human research separated by a dashed horizontal line. Reduction in Aβ refers to a reduction in the FUS-targeted regions unless mentioned otherwise. AD: Alzheimer’s disease; MRgFUS: MRI-guided focused ultrasound; Aβ: amyloid-β; Tg: transgenic; GSK: glycogen synthase kinase; SUS: scanning ultrasound; TrkA: Tropomyosin receptor kinase A; IVIg: intravenous immunoglobulin; BBB: blood-brain barrier; PDD: Parkinson’s disease dementia; PET: positron emission tomography; MCI: mild cognitive impairment.

**Supplementary Table 2**: Summary of the reviewed sources for section 2.2 Receptor-mediated transcytosis (RMT)

| **Authors** | **BBB target receptor** | **Drug type** | **Population** | **Results** |
| --- | --- | --- | --- | --- |
| Barker et al (2023) | Tfr^mu/hu^ (human) | ASO | C57BL/6 mice and NHP | More uniform ASO distribution compared to direct ASO delivery in CSF |
| Hammond et al (2022) | Tfr (murine) | ASO | SMA mouse model | Increases ASO biodistribution in CNS |
| Rofo et al (2022) | Tfr (murine) | NEP | Tg-ArcSwe mice | Increases NEP brain uptake |
| Campos et al (2020) | Tfr (murine) | NEP | Non-Tg rats | Increases CSF exposure of NEP and reduces Aβ in CSF and brain parenchyma |
| Ullman et al (2020) | Tfr^mu/hu^ (human) | Iduronate 2-sulfatase (IDS) | IDS KO mice | Improves brain delivery of IDS |
| Wuensche et al (2022) | Tfr (murine) | Anti-Aβ antibody (Aducanumab) | APP/PS1 mice | DFO* chelator is essential for ^89^Zr-immuno-PET imaging |
| Stergiou et al (2023) | Tfr (murine) | Anti-Aβ antibody (Aducanumab) | APP/PS1 mice | Increases antibody brain delivery |
| Hultqvist et al (2017) | Tfr (murine) | Anti-Aβ antibody (mAb158) | Tg-ArcSwe mice | Tfr shuttle (with monovalent Tfr binding) increases brain delivery of antibody |
| Fang et al (2019) | Tfr (murine) | Anti-Aβ antibody (3D6) | Tg-Swe and Tg-ArcSwe mice | A small bispecific antibody-based PET radioligand shows affinity to fibrillary and soluble Aβ and has shorter blood half-life compared to other bispecific antibodies |
| Niewoehner et al (2014) | Tfr (murine) | Anti-Aβ antibody (mAb31) | PS2APP mice | Increases Aβ target engagement, with monovalent binding to Tfr important for transcytosis |
| Syvänen et al (2018) | Tfr (murine) | Anti-Aβ antibody (mAb158) | Tg-ArcSwe mice | Causes more widespread antibody brain distribution and more localized to Aβ rich brain regions |
| Gustavsson et al (2023) | Tfr (murine) | Anti-Aβ antibody (mAb158) | APP^NL-G-F^ mice | Tfr shuttle does not increase the therapeutic benefit of anti-Aβ antibody |
| Kariolis et al (2020) | Tfr^mu/hu^ (human) | Anti-BACE1 and anti-tau antibodies | WT mice and NHP | Increases brain concentration of antibody in mice and monkeys |
| Yu et al (2014) | Tfr (primate) | BACE1 antibody | WT mice and NHP | Increases antibody brain penetrance; degree of brain Aβ reduction is correlated with brain concentration of anti-Tfr/BACE1 antibody |
| Grimm et al (2023) | Tfr (human) | Anti-Aβ antibody (Gantenerumab) | NHP | Increases brain exposure of antibody and causes a more homogeneous distribution compared to non-Tfr antibody |
| Baodo et al (2010) | IR (human) | Anti-Aβ antibody | NHP | Tfr-targeted antibody is rapidly cleared from plasma and reaches the primate brain |
| van Lengerich et al (2023) | Tfr^mu/hu^ (human) | TREM2 antibody | APP^SAA^, TREM2 BAC Tg, and 5xFAD mice | Improves brain exposure and activity of TREM2 antibody |
| Zuchero et al (2016) | CD98hc (murine); basigin, Glut1 (murine); and chimeras | BACE1 | WT mice | CD98hc shuttle increase antibody brain uptake and reduces Aβ |
| Pornnoppadol et al (2024) | CD98hc (murine); Tfr (murine) | IgG | WT mice | CH98hc have longer-lived brain retention of IgGs compared to Tfr-targeted shuttles |
| Yu et al (2011) | Tfr (murine) | BACE1 | WT mice | Low affinity binding between anti-Tfr antibodies and Tfr results in increased brain delivery |
| Johnsen et al (2019) | Tfr (murine) | Oxaliplatin / Anti-Tfr antibodies on nanoparticles | In vitro BBB model and non-Tg mice | High density anti-Tfr antibodies on surface of nanoparticle results in increased transport; off-target accumulation in spleen by Tfr targeting |
| *CTAD* (2023) | Tfr (human) | Anti-Aβ antibody (Gantenerumab --> Trontinemab) | Prodromal or mild-to-moderate AD | Aβ removal is achieved at lower doses than with typical mAbs |
| *News release: Denali Therapeutics Reports Second Quarter 2023 Financial Results* | Tfr (human) | TREM2 agonist | Healthy volunteers | DNL919 clinical development is discontinued after it a phase 1 study showed signs of narrow therapeutic window |

Preclinical/animal research and human research separated by a dashed horizontal line. Tfr: transferrin receptor; ASO: antisense-oligonucleotide; CNS: central nervous system; NHP: non-human primates; SMA: spinal muscular atrophy; NEP: neprilysin; KO: knock-out; WT: wild-type; Aβ: amyloid-β; BACE1: β-secretase 1; IR: insulin receptor; AD: Alzheimer’s disease; BBB: blood-brain barrier.

**Supplementary Table 3**: Summary of the reviewed sources for section 2.3 Nanoparticle-based carriers

| **Authors** | **Nanoparticle** | **Drug** | **Cell type / population** | **Results** |
| --- | --- | --- | --- | --- |
| Pinheiro et al (2020) | Lipid nanoparticles (2 types both with Tfr functionalization) | Quercetin | hCMEC/D3 cell line | Tfr functionalization of both nanoparticles does not significantly increase BBB permeability |
| Topal et al (2020) | Solid lipid nanoparticles | Donepezil | Primary rat brain cell cultures; hCMEC/D3 cell line | ApoE targeting of nanoparticles results in increased uptake across cell cultures |
| Rotman et al (2015) | Liposomes (2 types) | Anti-Aβ antibody (fragments) | APP/PS1 mice | Both types of liposomes increased the brain delivery of drug encapsulated liposomes, though in varying degrees |
| Wilson et al (2008) | Poly(n-butylcyanoacrylate) nanoparticles | Rivastigmine | Non-Tg rats | Nanoparticles coated with polysorbate 80 results in higher brain concentration |
| Carradori et al (2018) | Polymer nanoparticles | Anti-Aβ 1-42 | WT and Tg mice | Nanoparticles reduced memory deterioration and reduced soluble amyloid levels |
| Badr et al (2024) | Lipid nanoparticles | miR-17 inhibitor | WT and 5xFAD mice | Anti-17 MNLPs (mannose lipid nanoparticles) reduced amyloid burden and neuroinflammation and reduced spatial memory deterioration |
| Rajput et al (2022) | Liposomes | Donepezil | Sheep nasal mucosa; non-Tg rats | Nasal administration of liposomes enhances brain concentration |
| Al Harthi et al (2019) | Liposomes | Donepezil | Sheep nasal mucosa; non-Tg rabbits | Nasal administration of liposomes enhances brain concentration |
| *Clinical trial: Study of APH-1105 in Patients With Mild to Moderate Alzheimer's Disease* *(2021)* | Unknown, for intranasal administration | APH-1105 (α-secretase modulator) | Mild-to-moderate AD | Ongoing |

Preclinical/animal research and human research separated by a dashed horizontal line. Aβ: amyloid-β; hCMEC/D3: human cerebral microvascular endothelial cell line; BBB: blood-brain barrier; Tg: transgenic; ApoE: apolipoprotein E; AD: Alzheimer’s disease
